# Case report: Preliminary study on the diagnosis and treatment of respiratory distress in patients with giant nodular goiter complicated with severe COVID-19

**DOI:** 10.3389/fmed.2023.1204658

**Published:** 2023-09-06

**Authors:** Fang Wang, Xing Yu, Zhangxia Ren, Yong Wang

**Affiliations:** ^1^Department of Thyroid and Breast Surgery, Guang'an People's Hospital, Guang'an, Sichuan, China; ^2^Department of Thyroid Surgery, The Second Affiliated Hospital of Zhejiang University School of Medicine, Hangzhou, Zhejiang, China

**Keywords:** giant nodular goiter, COVID-19, respiratory distress, diagnosis and treatment plan, surgery

## Abstract

**Background:**

To investigate the practicality of emergency surgical and conservative medical treatments in patients with giant nodular goiter complicated by severe coronavirus disease 2019 (COVID-19)-related respiratory distress, evaluate the prognosis based on the two interventions, and explore the diagnosis and treatment plan of COVID-19-related respiratory distress in patients with giant nodular goiter.

**Methods:**

Four cases were retrospectively collected. Among them, two cases underwent emergency surgery, one case was treated with conservative treatment, whereas the fourth case underwent emergency surgery after failure of conservative therapy.

**Results:**

Dyspnea was significantly improved postoperatively, and the endotracheal tube was successfully removed 10.5 h after the operation, but inflammatory markers were greatly enhanced as compared to the preoperative values, patients with different degrees of fever, cough, and other discomforts postoperatively. Case 1 showed complete remission of all symptoms after 3 weeks, while case 2 displayed fever, cough, drowsiness, and other symptoms after the discharge and was eventually readmitted. In case 3, the conservative COVID-19 treatment marginally improved the pulmonary infection, fever, and other symptoms, but cough and other discomforts were persistent, along with delirium in later stages. Moreover, case 4 reported extubation failure after undergoing treatment with the standard new coronary pneumonia regimen in the tracheal intubation state; however, the patient was successfully weaned and extubated 9 days after emergency surgery to relieve the obstruction.

**Conclusion:**

Our preliminary exploration suggested that patients with giant nodular goiter and respiratory tract obstruction post-acute COVID-19 infection can undergo early surgery after surgical tolerance evaluation for a better prognosis.

## Introduction

Since the outbreak of novel coronavirus disease 2019 (COVID-19), which is caused by SARS-CoV-2, several COVID-19-related thyroid diseases have emerged, including subacute thyroiditis, autoimmune thyroid disorders, non-thyroidal illness syndrome, and thyroid dysfunction due to unknown causes (1–3). Although a few respiratory distress cases have been recorded in which patients presented with tracheal compression due to giant nodular goiter superimposed with novel COVID-19 infection, only one case has been reported by Amin et al. (4). Moreover, it is still unknown whether novel COVID-19 infection can aggravate the tracheal compression symptoms and whether tracheal compression in giant nodular goiter cases can lead to prolonged COVID-19 pneumonia, which is difficult to treat. Furthermore, the efficacy of emergency tracheal intubation, ventilator-assisted support, or emergency thyroidectomy for relieving tracheal obstruction or a conservative treatment after controlling pulmonary infection in patients with dyspnea is still ambiguous. Does an emergency surgery increase the severity of lung infection and leads to enhanced inflammatory markers? Does the conservative treatment delay the optimal timing of surgery? How does an extended tracheal intubation time result in tracheomalacia, extubation failure, etc.? A precise evaluation of the need for surgical or conservative treatment is a key factor governing our current clinical decisions. Combined with the experience of treating four giant nodular goiter cases having tracheal compression coupled with severe coronavirus pneumonia post-COVID-19 pandemic in *The Second Affiliated Hospital Zhejiang University School of Medicine*, our study has discussed the development of an individualized treatment plan for emergency surgery adapted to the general population as mentioned below.

## Case introduction

### Inclusion criteria

Patients with giant nodular goiter with tracheal compression and superimposed severe COVID-19 infection with accompanying symptoms.

### Clinical data

This study was approved by the Human Research Ethics Committee of the Second Affiliated Hospital of Zhejiang University, and obtained the patient’s consent.

Case 1: A 24-year-old female patient, after reporting dyspnea for >3 h and fever, with the highest body temperature of 37.9°C, without any cough or expectoration, was admitted on December 16, 2022. Physical examination showed deep breathing, respiratory rate 40 breaths/min, cyanotic lips and nail bed, clear breath sounds in both lungs, and no obvious rales. She had a previous history of hearing loss, while the remaining findings were unremarkable. After admission, the COVID-19 antigen test was positive, whereas the oxygen saturation was 86% (reference value 95–100%), white blood cell (WBC) 9.7 × 10^9^/L (reference value 4–10 × 10^9^/L), neutrophil percentage 76.4% (reference value 50.0–70.0%), c-reactive protein (CRP) 1.7 mg/L (reference value<10.0 mg/L), and interleukin (IL)-6133.50 pg/mL (reference value<7.0 pg/mL). An emergency computed tomography (CT) revealed marked bilateral thyroid enlargement with uneven density, compression, and tracheal as well as esophageal narrowing, with no significant positive chest CT signs. Thyroid hormone level: TT3 1.11 nmol/L (reference value 0.98–2.33), TT4 70.5 nmol/L (reference value 62.7–150.8), FT3 3.27 pmol/L (reference value 2.43–6.01), FT4 11.45 pmol/L (reference value 9.01–19.05), TSH 0.29 mIU/L (reference value 0.35–4.49), TPOAB 3.38 IU/mL (reference value<5.61), ATGAB 1.20 IU/mL (reference value < 4.11), TG >500 μg/L (reference value 3.50–77.00), Diagnostic considerations: novel coronavirus pneumonia (severe). After a multidisciplinary consultation, and excluding dyspnea caused by cardiopulmonary function, “bilateral total thyroidectomy (retrosternal thyroidectomy)” was performed under general anesthesia with endotracheal intubation at 00: 10 AM on December 17, 2022. The operative time was 1 h and 35 min. Subsequently, the patient was shifted to ICU postoperatively and was provided assisted ventilation. The patient was successfully extubated 10 h after the operation and transferred to the isolation ward after becoming stable. One day after the operation, the patient developed a fever, with the highest temperature of 39.1°C. WBC count was 7.8 × 10^9^/L (reference value 4–10 × 10^9^/L), neutrophil percentage was 79.6%L (reference value 95–100%), CRP was 30.8 pg/mL, procalcitonin (PCT) was 1.52 ng/mL (reference value<0.5 ng/mL), and IL-6 was 30.8 pg/mL. The patient got relief after symptomatic cooling treatment. As there was no cough, expectoration, muscle soreness, or any other discomfort during hospitalization, the patient was discharged 3 days postoperatively. A postoperative pathological examination showed thyroid nodules in the bilateral lobes of the thyroid gland. Postoperative follow-up was: The patient reported cough and expectoration after discharge, which was completely relieved after 3 weeks, without dyspnea, fever, fatigue, or other discomforts. Currently, the patient is in good physical condition.

Case 2: A 74-year-old female patient was admitted due to dyspnea for >1 h on December 19, 2022, without any fever, cough, expectoration, etc. Physical examination: dysphoria, extreme dyspnea, cyanotic lips and nail bed, respiratory rate 40 breaths/min, clear breath sounds in both lungs, no obvious rales heard. Being a deaf-mute patient, she had a history of hypertension and right partial thyroidectomy >20 years ago, a COVID-19 positive antigen test, oxygen saturation of 67%, and emergency tracheal intubation. The auxiliary examination showed WBC as 5.8 × 10^9^/L, neutrophil percentage 74.6%, CRP 11.0 mg/mL, PCT 0.44 ng/mL, and IL-6 20.40 pg/mL. An emergency CT showed bilateral thyroid enlargement with multiple nodules and necrotic areas, right airway stenosis, throat edema, pleural effusion, and thoracic cavity effusion. Thyroid hormone level: TT3 1.33 nmol/L (reference value 0.98–2.33), TT4 74.7 nmol/L (reference value 62.7–150.8), FT3 3.87 pmol/L (reference value 2.43–6.01), FT4 9.47 pmol/L (reference value 9.01–19.05), TSH 1.56 mIU/L (reference value 0.35–4.49). Diagnostic considerations: novel coronavirus pneumonia (critical type). After a multidisciplinary consultation, cardiopulmonary function-related dyspnea was excluded. On December 19, 2022, the patient underwent “left total thyroidectomy + right partial thyroidectomy” under general anesthesia with endotracheal intubation. The operative time was 1 h and 49 min. The patient was admitted to ICU postoperatively and given assisted ventilation. Furthermore, 11 h later, the patient was successfully extubated and transferred to the general ward. The patient was given anti-infection drugs, fluid infusion, and supportive treatment. One day after the operation, the patient developed a fever, with a body temperature of 38.4°C, shortness of breath with a rate of about 40 breaths/min, and oxygen saturation of 93%. The patient was immediately given nasal catheter oxygen inhalation of 3–5 L/min. Laboratory investigations revealed the WBC count as 12.6 × 10^9^/L, neutrophil percentage 90.4%, CRP 97.1 mg/mL, and PCT 0.65 ng/mL. Based on this, the patient underwent dexamethasone intravenous bolus, anti-infection, atomization inhalation, phlegm reduction, and other symptomatic treatment. The vital signs were stable 3 days after the operation, without any recurrence of shortness of breath, fever, and oxygen saturation reduction. Although the patient was discharged 3 days postoperatively, the postoperative pathological examination revealed a nodular goiter of the left thyroid gland and isthmus with hemorrhagic cystic degeneration and focal follicular epithelial dysplasia. For right thyroid nodular goiter, 2 days after discharge, the patient displayed repeated fever (maximum body temperature: 38.5°C), fatigue, drowsiness, cough, and expectoration, without dyspnea. Subsequently, the patient was hospitalized in a local hospital and discharged after an improvement.

Case 3: An 89-year-old female patient was admitted due to a cough for >3 days on December 28, 2022. On admission day, the patient was diagnosed with a new COVID-19 infection, significant cough, and expectoration, respiratory rate 40 breaths/min, cyanotic lips and nail bed, without fever, and moist rales could be heard in both lungs. The patient also had a history of previous thyroid surgery, hypertension, and Parkinson’s disease. After the patient visited our emergency department, the investigations revealed oxygen saturation of 92%, WBC 3.0 × 10^9^/L, neutrophil percentage of 78.1%, CRP 5.3 mg/mL, PCT 0.04 ng/mL, and IL-6 27.3 pg/mL. A CT scan suggested bilateral thyroid enlargement, right deviation of tracheal compression, and scattered cord shadows, as well as exudations in both lungs, pleural effusion. Thyroid hormone level: TT3 1.34 nmol/L (reference value 0.98–2.33), TT4 88.9 nmol/L (reference value 62.7–150.8), FT3 4.07 pmol/L (reference value 2.43–6.01), FT4 12.00 pmol/L (reference value 9.01–19.05), TSH 0.32 mIU/L (reference value 0.35–4.49), TPOAB 4.22 IU/mL (reference value<5.61), ATGAB 9.60 IU/mL (reference value < 4.11), TG >500 μg/L (reference value 3.50–77.00). Diagnostic considerations: novel coronavirus pneumonia (severe). After admission, the patient was given oxygen inhalation, nutritional support, oral antiviral therapy with nelmativir+ritonavir, anti-infection drugs, atomization inhalation, phlegm reduction, anticoagulation drugs, and other symptomatic treatment. By the end of the study, after >50 days of hospitalization, the patient’s lung infection, fever, and other symptoms were marginally improved, but cough and other discomforts, as well as delirium and other psychiatric symptoms at the later stages, were still observed. Furthermore, the patient was still hospitalized by the time our study’s deadline ended.

**Figure 1:**
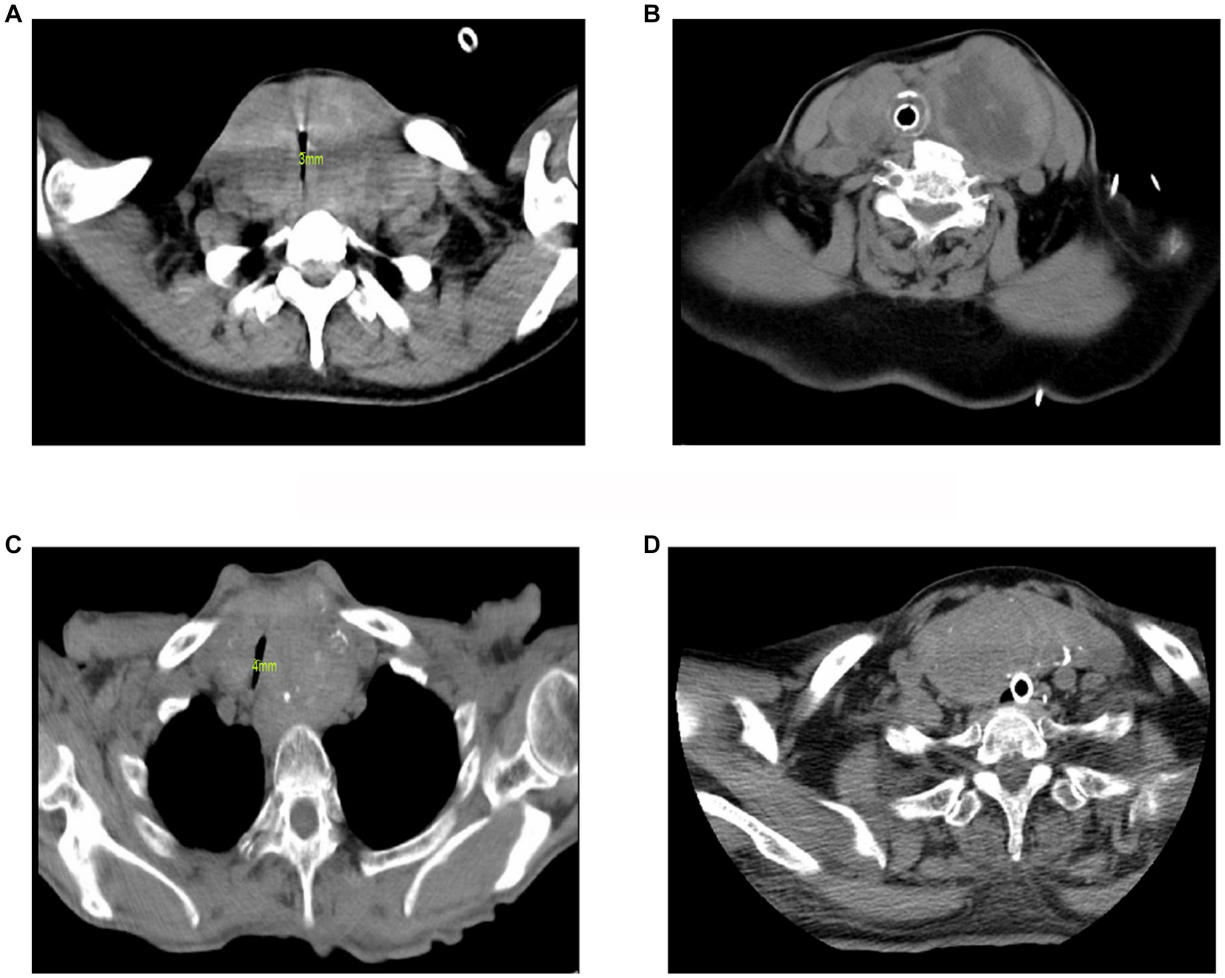
Preoperative cervical CT images of 4 cases. **(A)** Case 1. The size of the left thyroid gland was 44 × 46 mm, and the size of the right thyroid gland was 40 × 28 mm. Protruding behind the sternum, the trachea was significantly compressed, and the diameter of the narrowest part was 3 mm. **(B)** Case 2. Under tracheal intubation, the trachea moved to the right, the size of the left thyroid gland was about 61 × 60 mm, and the size of the right thyroid gland was about 41 × 24 mm. **(C)** Case 3. The size of the left thyroid gland was about 79 × 65 mm, the size of the right thyroid gland was about 64 × 41 mm, the trachea was shifted to the right, and the diameter of the narrowest part was 4 mm. **(D)** Case 4. Under tracheal intubation, after the left partial thyroidectomy, the size of the right thyroid gland was about 87 × 38 mm, protruding behind the sternum.

**Figure 2:**
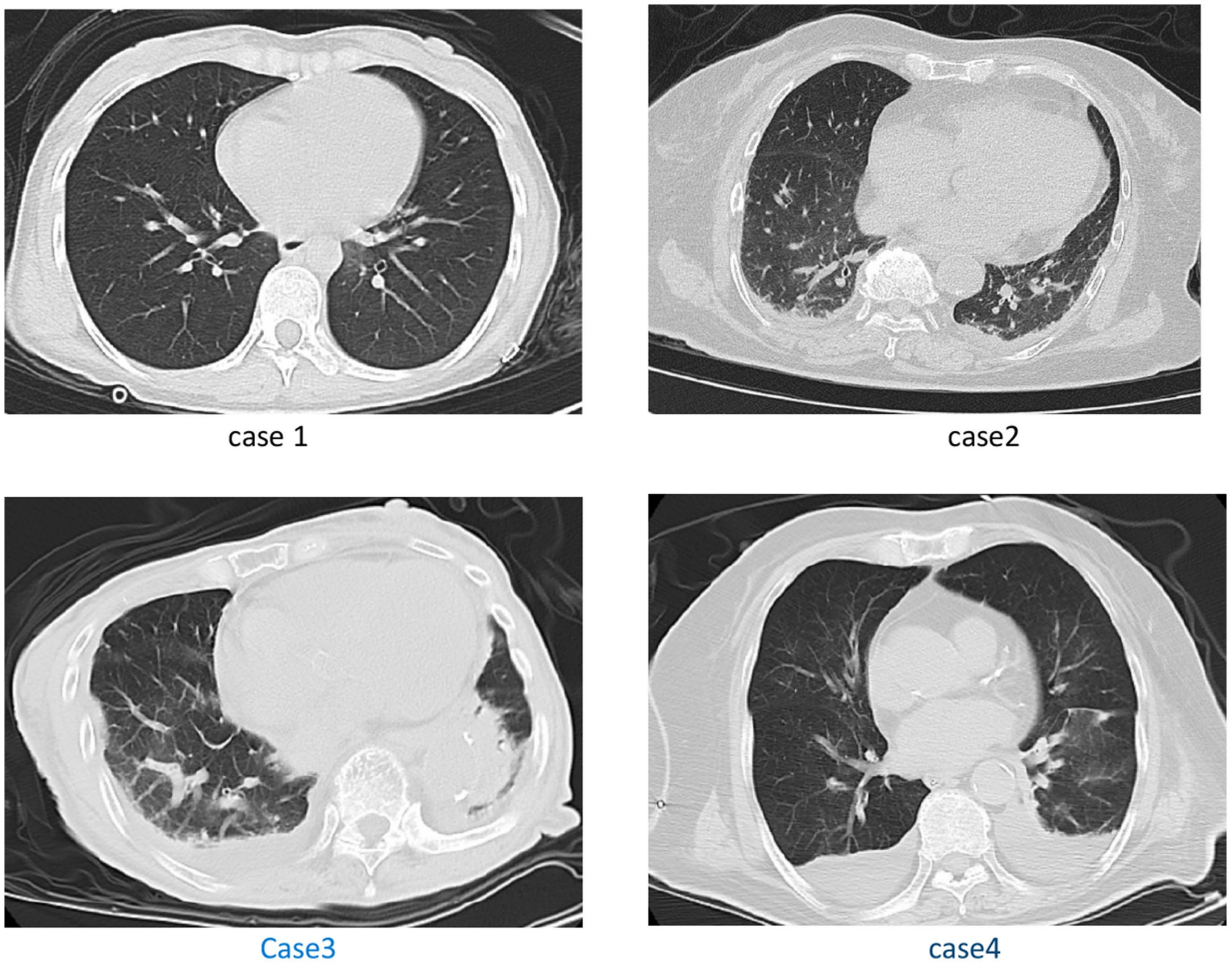
Case 1: with no significant positive chest CT signs. Case 2: pleural effusion, and thoracic cavity effusion. Case 3: scattered streak shadows in bilateral lung, scattered exudation in bilateral lung, pleural effusion. Case 4: multiple patchy high-density shadows in both lungs, and bilateral pleural effusion with adjacent lung tissue atelectasis.

Case 4: An 84-year-old female patient with airway foreign body obstruction for >2 weeks was admitted to our hospital on January 11, 2023. Having a history of left partial thyroidectomy, she reported chest tightness, shortness of breath, and cyanotic lips after eating the “pill” >2 weeks ago and visited a local hospital for an oxygen saturation level of 50%. She underwent emergency endotracheal intubation, airway clearance, and foreign body aspiration, while her oxygen saturation was maintained at >95%. An emergency CT revealed possible local compression of retrosternal goiter, multiple patchy high-density shadows in both lungs, and bilateral pleural effusion with adjacent lung tissue atelectasis. After admission, her COVID-19 antigen test was positive; however, her general condition improved after the administration of anti-infection, hormonal, and anti-inflammatory drugs with other treatments. Three days later, the removal of tracheal intubation further decreased the patient’s oxygen saturation level to 70%. The tracheal intubation was performed again. Seven days post-admission, the patient maintained oxygen saturation > 93% under endotracheal intubation and spontaneous breathing. After the removal of the endotracheal tube, the oxygen saturation decreased to about 70%, 1.5 h after observation. The patient came to our hospital for emergency treatment after second endotracheal intubation. Physical examination showed sedation, endotracheal intubation, ventilator-assisted breathing, and crackles heard in both lungs. Emergency CT revealed thyromegaly with less uniform density, tracheal compression, two pulmonary inflammatory exudates, partial right upper lung consolidation, and bilateral pleural effusion with adjacent lung tissue atelectasis. Her investigations revealed WBC count as 17.8 × 10^9^/L, neutrophil percentage 93.6%, CRP 37.8 mg/mL, PCT 0.35 ng/mL, IL-6 19.7 pg/mL, and albumin 22.5 g/L. Thyroid hormone level: TT3 1.77 nmol/L (reference value 0.98–2.33), TT4 65.6 nmol/L (reference value 62.7–150.8), FT3 2.98 pmol/L (reference value 2.43–6.01), FT4 15.85 pmol/L (reference value 9.01–19.05), TSH 0.03 mIU/L (reference value 0.35–4.49), TPOAB <0.5 IU/mL (reference value < 5.61), ATGAB 6.67 IU/mL (reference value<4.11), TG 99.81 μg/L (reference value 3.50–77.00). Diagnostic considerations: novel coronavirus pneumonia (critical type). After admission to ICU, the patient was given assisted ventilation, nutritional support, anti-infection drug, atomization inhalation, expectorant, anticoagulant, correction of hypoproteinemia, and other symptomatic treatment. On January 18, 2023, the patient underwent “retrosternal right thyroidectomy and isthmectomy+left thyroidectomy” under emergency general anesthesia with an operation time of 2 h and 10 min. The patient was admitted to the ICU postoperatively and given assisted ventilation, an anti-infection drug, fluid infusion, and symptomatic treatment. After trying to remove the endotracheal tube 6 days after the operation, the patient developed three concave signs (refers to significant depression of the suprasternal fossa, supraclavicular fossa, and intercostal space during inspiration when obstructive ventilatory dysfunction occurs), with profuse sweating, a decrease in oxygen saturation, and other symptoms. Moreover, the patient underwent endotracheal intubation and assisted ventilation again. Fiberoptic bronchoscopy revealed poor vocal cord mobility and marked swelling. Corticosteroids were administered to improve airway edema along with anti-infection drugs, nutritional support, and other treatments. The patient was successfully extubated 9 days postoperatively. After extubation, the patient displayed no obvious hoarseness, dyspnea, or other symptoms. The oxygen saturation level was 98% at rest. The patient was transferred to a local hospital 10 days after the operation to continue rehabilitation. A postoperative pathological examination showed bilateral thyroid and isthmic nodular goiter with calcification, collagenization, and hemorrhagic cystic degeneration. Ten days after the discharge, postoperative follow-up revealed that the patient experienced smooth breathing, oxygen saturation of 97–98% at rest, well-controlled pulmonary infection, intermittent delirium, and other psychiatric symptoms (Tables 1–4; Figures 1, 2).

**Table 1:** Inflammatory markers of 4 cases.

	WBC	Neutrophil percentage	CRP	IL-6	PCT
Case 1					
Preoperative	9.70×10^9/L	76.4%	1.7 pg/mL	133.50 pg/mL	(−)
Postoperative	7.80×10^9/L	79.6%	30.8 pg/mL	30.8 pg/mL	1.52 ng/mL
Case 2					
Preoperative	5.81×10^9/L	74.6%	11.0 pg/mL	20.40 pg/mL	0.44 ng/mL
Postoperative	12.60×10^9/L	90.4%	97.1 pg/mL	(−)	0.65 ng/mL
Case 3	3.00×10^9/L	78.1%	5.3 pg/mL	27.3 pg/mL	0.04 ng/mL
Case 4	17.80×10^9/L	93.6%	37.8 pg/mL	19.7 pg/mL	0.35 ng/mL
Reference value	4–10×10^^9^/L	50.0–70.0%	<10.0 mg/L	<7.0 pg/mL	0.5 ng/mL

WBC, white blood cell; CRP, C reactive protein; IL-6, Interleukin- 6; PCT, procalcitonin.

**Table 2:** Clinical presentation and treatment summary of 4 cases.

	Thyroid size	Tracheal diameter at narrowest point	Chest computed tomography	Surgery or not	Thyroid hormone levels	Oxygen saturation	Symptoms and signs	Inflammatory markers
Case 1	Left 44 × 46 mm Right 44 × 46 mm	3 mm	No positive findings	Yes	Table 4	Table 3		Table 1
Case 2	Left 61 × 60 mm Right 41 × 24 mm	–	Pleural effusion, and thoracic cavity effusion	Yes				
Case 3	Left 79 × 65 mm, Right 64 × 41 mm	4 mm	Scattered streak shadows, scattered exudation, pleural effusion					
Case 4	Left thyroidectomy, Right 87 × 38 mm	–	Patchy high-density shadows, bilateral pleural effusion with multiple adjacent lung tissues atelectasis	Yes				

**Table 3:** Thyroid hormone levels.

	FT3	FT4	TSH	TG	TPOAB	ATGAB	TT3	TT4
Case 1	3.27	11.45	0.29	>500	3.38	1.20	1.11	70.5
Case 2	3.87	9.47	1.56	–	–	–	1.33	74.7
Case 3	4.07	12.00	0.32	>500	4.22	9.60	1.34	88.9
Case 4	2.98	15.85	0.03	99.81	<0.5	6.67	0.77	65.6
Reference value	2.43–6.01 (pmol/L)	9.01–19.05 (pmol/L)	0.35–4.49 mIU/L	3.50–77.00 Ug/L	<5.61 IU/mL	<4.11 IU/mL	0.98–2.33 (nmol/L)	62.7–150.8 (nmol/L)

**Table 4:** Symptoms, signs, severity grade of COVID-19.

	Symptoms	Signs	Oxygen saturation	Novel coronavirus pneumonia grade
Case 1	Deep breathing, respiratory rate 40 times/min, lips and nail bed cyanosis	T 37.9°C Clear breath sounds in bilateral lung, without obvious rales	86%	Severe
Case 2	Dysphoria, extreme dyspnea, cyanotic lips and nail bed respiratory rate 40 breaths/min	No fever, clear breath sounds in bilateral lung, without obvious rales	67%	Critical type
Case 3	Cough, expectoration, shortness of breath 30 beats/min, cyanotic lips and nail bed	No fever, moist rales palpable in bilateral lung	92%	Severe
Case 4	Ventilator-assisted breathing status at admission to our hospital	Crackles heard in bilateral lung	50%	Critical type

Grading	Clinical presentation
Mild	
Mild clinical symptoms, no imaging findings of pneumonia	
Common type	
With fever, respiratory tract and other symptoms, imaging showed pneumonia	
Severe	Meet any of the following: Respiratory distress, RR ≥ 30 beats/; Oxygen saturation ≤ 93% at rest; Arterial partial pressure of oxygen (PaO2)/oxygen concentration (FiO2) ≤ 300 mmHg; Progressive aggravation of clinical symptoms, pulmonary imaging showed that the lesion significantly progressed >50% within 24–48 h
Critical type	Patients who meet one of the following conditions: Respiratory failure, and need mechanical ventilation; Shock; Combined with other organ failure need ICU monitoring treatment.

The grading criteria for COVID-19 in China.

## Discussion

COVID-19 infection is caused by a novel severe acute respiratory syndrome coronavirus 2 (SARS-CoV-2) and is mainly transmitted by the spread of respiratory droplets, with an incubation period of generally 3–7 days, usually ≤14 days. The main clinical manifestations are fever, fatigue, dry cough, respiratory failure, septic shock, and/or multiple organ dysfunction, and failure in severe cases, thus, endangering the patient’s life (5). On April 10, 2020, the World Health Organization (WHO) characterized COVID-19 as a global epidemic, as the infection involved >210 countries/regions (6). According to a WHO estimate, >670 million people worldwide have been infected, with >6.7 million deaths, however, 14–17% of infected patients may develop COVID-19-related acute respiratory distress syndrome (7), and 2.3% of them require endotracheal intubation intervention (8). However, Piazza et al. reported that long-term endotracheal intubation in COVID19 patients, might lead to tracheomalacia, stenosis, post-intubation granuloma, and tracheoesophageal leakage. Additionally, a tracheotomy performed 7–14 days after endotracheal intubation significantly enhances successful weaning rates and reduces associated complications as well as mortality as compared to long-term maintenance endotracheal intubation (9).

Giant nodular goiter is mostly caused by nodular goiter or thyroid adenoma, which can compress, shift and narrow the cervical trachea, thus, leading to ventilatory dysfunction. The incidence of tracheal compression and dyspnea caused by giant nodular goiter is 10–50%, while the incidence of dyspnea caused by retrosternal goiter is ≥75% (10). In the initial stage, the patient has no obvious discomfort and can experience dyspnea, along with other associated symptoms, whenever there is an increased oxygen demand in the case of long-distance exercise or weather changes. Furthermore, the probability of dyspnea significantly increases if such patients are suffering from COVID-19. So the question in these cases is whether COVID-19 infection or thyroid enlargement compressing the trachea is the main cause of respiratory distress. We believe that the respiratory distress observed in our cases was the result of the interplay between obstructive ventilatory dysfunction caused by compression from the giant nodular goiter and diffuse ventilatory dysfunction caused by COVID-19. We hypothesize that if other severe viral pneumonias were substituted, similar outcomes would be observed, given the persistence of obstructive ventilatory dysfunction caused by the goiter compression and diffuse ventilatory dysfunction caused by pneumonia. According to the clinical manifestations, examination, and imaging data of four patients, case 1 presented with low inflammatory indicators and no infection findings on pulmonary imaging. Since cervical CT showed that the diameter of the narrowest part of the trachea was 3 mm, dyspnea may be caused by an upper respiratory tract obstruction. Case 2 examination after endotracheal intubation revealed exudative bilateral pleural effusions, along with increased CRP and IL-6 values and unknown airway stenosis. In case 3 lung exudation, IL-6 increased significantly, while the diameter of the narrowest part of the trachea was 4 mm. Moreover, in case 4, examination after tracheal intubation revealed two pneumonic exudates, elevated inflammatory markers, and airway stenosis of unknown etiology. In COVID-19, the human cells are primarily attacked through angiotensin-converting enzyme 2 (ACE2) to induce an inflammatory response. Although ACE2 is expressed in the nose, oral mucosa, throat, and tongue, edema manifestations in the corresponding organs might occur (11–13). Another study by Tasnuva disclosed that dyspnea in giant nodular goiter with new COVID-19 pneumonia may be related to airway edema, thus, aggravating tracheal stenosis (4), and our findings in case 1 confirmed this view. However, we did not compare the relevant imaging data before the disease onset and did not perform a bronchoscopy to identify whether airway edema led to aggravated airway stenosis.

The Conservative management of the four cases is not the same, because we encountered numerous challenges during that period. In December 2022, China underwent a shift in the management and control of COVID-19. Consequently, a sudden surge in infections occurred, surpassing the preparedness of medical resources. There was a shortage of specific drugs, medical devices, and various supplies, making it difficult to ensure uniform treatment management for each patient during that time. Recent literature suggests that the time from COVID-19 onset to tracheal intubation intervention is usually 8–9 days (14, 15), while the mortality rate of critically ill patients is 16.7% ~ 61.5% (16, 17), of which the mortality rate at 24 h after tracheal intubation is 10.4%, and the 28-day mortality rate is ~61% (18). If dyspnea occurs in such patients, it requires tracheal intubation. Quick relief from respiratory tract obstruction and removal of tracheal intubation without delay is the key to improving the survival rate. While treating the four patients with giant nodular goiter complicated with severe COVID-19, we found that the dyspnea of patients undergoing emergency surgery significantly improved postoperatively, and the tracheal intubation was successfully removed 10.5 h after the operation on average. However, various inflammatory indicators postoperatively were significantly increased to different extents when compared with those before the operation, accompanied by different degrees of fever, cough, and other discomforts postoperatively. Since case 1 was younger, symptoms such as cough and sputum resolved completely after 3 weeks and recovered quickly after discharge, while case 2 was an elderly patient who was readmitted due to fever, cough, drowsiness, and other symptoms after discharge, however, emergency surgery in this patient resulted in aggravated inflammation. Although case 3 underwent the standard COVID-19 regime for long time after hospitalization, the patient’s lung infection, fever, and other symptoms were marginally improved, but cough and other discomforts, along with delirium and other psychiatric symptoms in later stages, were still observed. Case 4 failed multiple extubation attempts after the standard COVID-19 treatment regimen, which might have been caused by aggravated tracheal stenosis due to throat edema as a result of prolonged intubation time, patient lung inflammation causing lung dysfunction. The patient was successfully extubated 9 days after emergency surgery to relieve the obstruction. Ten days after discharge, the patient follow-up revealed smooth breathing and good control of pulmonary infection, but psychiatric symptoms such as delirium occurred in later stages. Additionally, psychiatric symptoms like delirium occurred in later stages in both the patients who underwent prolonged conservative treatment, which was either purely coincidental or might be related to other factors, but due to the small number of cases in our study, any relevant conclusions could not be made.

In patients requiring emergency surgery at our center, we perform preoperative emergency chest and neck CT as well as evaluation of various inflammatory indicators to assess tracheal compression, pulmonary inflammation, presence of non-recurrent laryngeal nerve, and immediate endotracheal intubation for patients showing significant reduction in oxygen saturation. During the operation, the operation time should be shortened to reduce the spread of inflammation along with precise ligation of the blood vessels to avoid second surgical trauma and protect the recurrent laryngeal nerve to avoid aggravated dyspnea caused by vocal cord injury and difficult extubation at a later stage. In order to shorten the operative time, such patients can undergo thyroidectomy with a large cervical incision, subtotal thyroidectomy, or near-total thyroidectomy.

After emergency tracheal intubation for patients with giant nodular goiter showing tracheal compression combined with an acute attack of severe COVID-19 with low oxygen saturation, the selection of the extubation and operation time is a crucial parameter. After 3–4 days of an acute attack, the tracheal intubation can be removed by reassessing the levels of inflammatory indicators and the patient’s general condition. If the extubation is smooth, symptomatic treatment can be continued or deferred to elective thyroidectomy later on. If such patients fail in multiple extubation attempts, like in our case number 4, and for patients with severe thyroid-related tracheal compression, emergency surgery can be performed simultaneously as anti-COVID-19 treatment after the general condition is corrected.

After a comparative analysis, we suggest the following advantages of emergency surgery: it can quickly relieve upper respiratory tract obstruction, and postoperative patients recover faster. However, this technique has a disadvantage, namely, surgery may aggravate the inflammatory response. Furthermore, using a conservative treatment can avoid surgical stimulation that might aggravate the patient’s inflammatory response. Although it has a few disadvantages: it may lead to extubation difficulties, as long-term intubation may lead to aggravation of pulmonary infection, tracheomalacia, and other complications, and even delirium and other psychiatric symptoms at the later stages. In contrast, emergency surgery in such patients can provide a better prognosis. For choosing the timing of surgery, we suggest that before the COVID-19 progresses to critical illness, early surgery should be performed when the function of other organs is not significantly impaired, the earlier the operation, the greater the benefit to patients. Amin et al. (4) also recommended that in nodular goiter patients with respiratory obstruction, a timely intervention to relieve airway obstruction is the best choice. By analogy, patients with other diseases that may lead to acute exacerbation of pulmonary inflammation (such as chronic obstructive pulmonary disease) superimposed with giant nodular goiter and tracheal compression may also give priority to surgical treatment.

## Conclusion

It is difficult to choose an accurate treatment plan for a giant nodular goiter with COVID-19-related respiratory tract obstruction. Our preliminary exploration suggests that an early surgery to relieve respiratory tract obstruction in such patients after evaluation of surgical tolerance can provide a better prognosis. However, due to fewer study cases in our center, this specific situation requires more samples for precise analysis to derive better conclusions.

## Data availability statement

The original contributions presented in the study are included in the article/supplementary material, further inquiries can be directed to the corresponding author.

## Ethics statement

The studies involving humans were approved by Human Research Ethics Committee of the Second Affiliated Hospital of Second Affiliated Hospital of Zhejiang University School of Medicine. The studies were conducted in accordance with the local legislation and institutional requirements. The participants provided their written informed consent to participate in this study. Written informed consent was obtained from the individual(s) for the publication of any potentially identifiable images or data included in this article. Written informed consent was obtained from the participant/patient(s) for the publication of this case report.

## Author contributions

FW, XY, ZR, and YW performed the material preparation, data collection, and analysis. FW wrote the first draft of the manuscript. All authors commented on previous versions of the manuscript, contributed to the study conception and design, read, and approved the final manuscript.

## Funding

This work was supported by the Zhejiang Basic Public Welfare Project (LGF22H070002); and the National Natural Science Foundation of China (82201265).

## Conflict of interest

The authors declare that the research was conducted in the absence of any commercial or financial relationships that could be construed as a potential conflict of interest.

## Publisher’s note

All claims expressed in this article are solely those of the authors and do not necessarily represent those of their affiliated organizations, or those of the publisher, the editors and the reviewers. Any product that may be evaluated in this article, or claim that may be made by its manufacturer, is not guaranteed or endorsed by the publisher.
